# Polymer Micelles as Nanocarriers of Bioactive Peptides

**DOI:** 10.3390/polym17091174

**Published:** 2025-04-25

**Authors:** Petar D. Petrov, Slavena Davidova, Galina Satchanska

**Affiliations:** 1 Institute of Polymers, Bulgarian Academy of Sciences, Akad. G. Bonchev St., Bl. 103A, 1113 Sofia, Bulgaria; 2 Department of Natural Sciences, New Bulgarian University, Montevideo Str. 21, 1618 Sofia, Bulgaria; stdavidova@nbu.bg (S.D.); gsatchanska@nbu.bg (G.S.); 3 UPIZ “Educational and Research Laboratory of Biology”-MF, New Bulgarian University, Montevideo Str. 21, 1618 Sofia, Bulgaria

**Keywords:** polymer micelles, nanocarriers, bioactive peptides, complexes

## Abstract

Bioactive peptides (BPs) have demonstrated diverse inhibitory effects against parasites, viruses, bacteria, fungi, and other pathogens, and therefore, they have been extensively used for developing various therapeutics. However, several challenges for the clinical use of BPs related to their stability, bioavailability, and cytotoxicity remain. The encapsulation of BPs in polymer micelles (PMs) has emerged as an effective strategy that can improve the pharmacological profiles, safety, and efficacy of treatments. This review describes the recent advances of micellar carriers of peptides with antimicrobial, anticancer, anti-inflammatory, immunomodulatory, and anti-diabetic activities. The mode of action of BPs and the unique characteristics of PMs are described, and a critical evaluation of their advantages and disadvantages is made. The upcoming challenges and future perspectives of micellar systems carrying BPs are discussed as well.

## 1. Introduction

**Figure 1:**
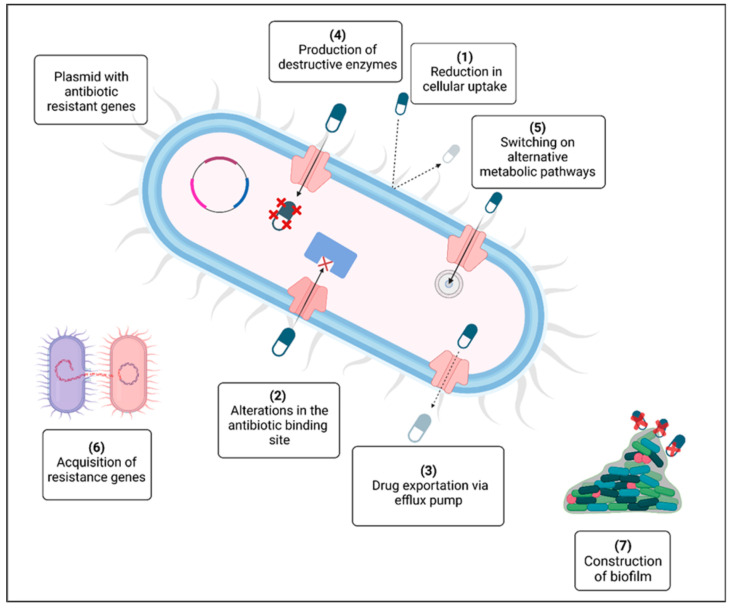
Mechanisms for the development of antibiotic resistance [1].

The emergence of antibiotic resistance in the past twenty years has become a major global danger to humankind [1]. The rise in multidrug-resistant pathogenic bacteria threatens the effectiveness of antibiotics, which have historically revolutionized medical science. The problem of antimicrobial resistance is associated with the inappropriate use of these drugs and the absence of new treatments stemming from strict regulatory requirements and diminished financial incentives. Figure 1 illustrates the various mechanisms that contribute to the emergence of antibiotic resistance in bacteria: (1) the breakdown of drugs through the production of inactivating enzymes; (2) a decrease in antibiotic uptake; (3) the alteration of a common metabolic pathway; (4) expulsion of the drug from the cell via an efflux pump; (5) modification of the antibiotic target; (6) the availability of a plasmid carrying multiple resistance genes; (7) the exchange of resistance genes among bacteria; and (8) the development of a protective extracellular polymeric matrix. The red crosses (X) indicate destructive enzymes. Significant efforts are necessary to impede the rise in resistance by studying new microorganisms, their resistance strategies, and diverse antimicrobial agents. A multidisciplinary strategy is needed throughout healthcare systems and the environmental and agricultural arenas. Antimicrobial peptides (AMPs) can serve as alternative therapeutic agents and may be crucial in combating antibiotic resistance [2].

Typically, AMPs, which can vary in size, consist of no more than 100 amino acid residues. They are integral to the innate immune defense in various organisms, including bacteria, fungi, plants, fish, invertebrates, amphibians, crustaceans, insects, reptiles, mammals, and humans. The majority of eukaryotic AMPs have cationic properties that define their characteristics. Additionally, they display both hydrophilic and lipophilic traits, classifying them as amphiphilic.

**Figure 2:**
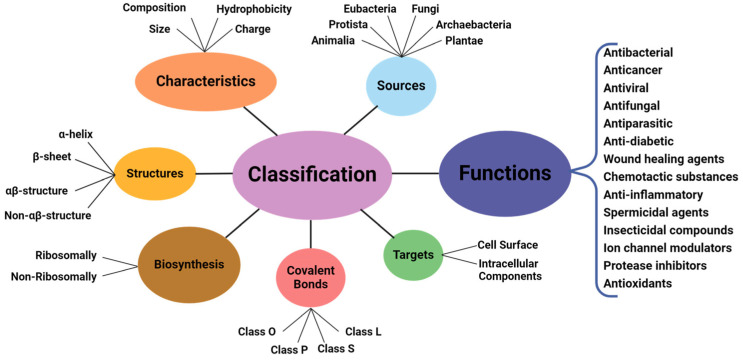
Classification of antimicrobial peptides [3].

The amphiphilic nature and cationic charge of these peptides allow them to attach to the negatively charged surfaces of pathogen cells, inserting into membranes to form pores and channels that eventually cause cell death. Figure 2 illustrates the classification of AMPs based on seven criteria [3]. The three-dimensional structural classification divides AMPs into four categories: alpha (α), beta (β), alpha-beta (α-β), and non-α-β. These categories are determined by the presence of helical structures, β-strands, a mix of helical and β-structures, or a lack of both types. In terms of covalent bond classification, peptides are sorted into four groups according to their polypeptide chain-bonding patterns: Class O includes circular peptides formed by a bond between the N-terminal and C-terminal backbone atoms; Class P features peptides shaped like the letter “P”, formed by a bond between one amino acid’s side chain and another’s backbone; Class S includes peptides with bonds between different side chains; and Class L encompasses all linear peptides.

Peptides, which come from natural sources, are biologically effective and are well tolerated when administered. They specifically bind to their receptors, which limits non-specific interactions with other body areas and helps avoid undesired side effects [4]. Nonetheless, several obstacles persist in the clinical application of antimicrobial peptides (AMPs) concerning their stability, bioavailability, and cytotoxicity.

**Figure 3:**
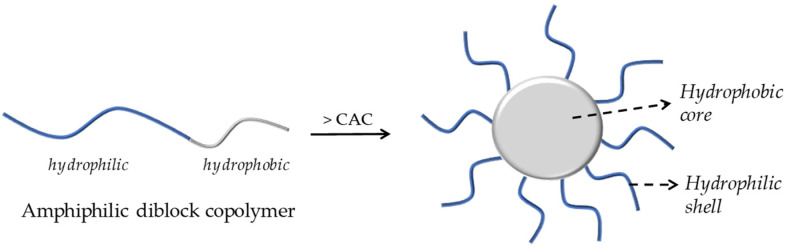
A schematic illustration of the formation of core–shell micelles by aggregation of an amphiphilic diblock copolymer.

Polymeric micelles have been widely researched for their ability to carry a wide range of biologically active compounds, including drugs and nucleic acids. Micellar nanocarriers, in particular, have shown promise in enhancing AMP therapies’ pharmacological profiles, safety, and effectiveness. PMs are usually formed through the self-assembly of an amphiphilic block or graft copolymers in an aqueous solution, resulting in colloidal particles, generally at the nanoscale, with a hydrophobic core and a hydrophilic shell, as illustrated in Figure 3 [5].

These micelles efficiently solubilize hydrophobic drugs within their core, providing excellent encapsulation efficiency, enhanced bioavailability, and controlled or targeted drug release. The micellar exterior safeguards the hydrophobic core from biological threats and can hold hydrophilic active components or nucleic acids. Furthermore, the specific micellar structure can be easily modified, enabling the design of multifunctional polymeric delivery systems. In this context, the design and use of polymeric micelles for transporting and delivering drugs and nucleic acids remains a vibrant research area experiencing ongoing development [6].

Micellar carriers have been utilized for drug delivery targeting breast cancer cells while minimizing the impact on healthy cells. These carriers are crucial in cellular uptake within drug delivery systems, ensuring effective medication delivery to the intended sites. By designing drug-interactive motifs, they function as an advanced drug carrier system, improving the efficacy and precision of drug delivery [7,8]. Their potential as therapeutics for osteosarcoma positions them as a promising area of research and development in cancer treatment. These carriers are essential for the targeted delivery of chemotherapeutic drugs, ensuring the medication effectively reaches cancerous cells [9,10]. They can also operate as a targeted delivery system to improve the delivery of antiviral drugs to CD4+ HIV host cells, maximizing the treatment’s impact. Furthermore, micelle-forming peptide amphiphiles display potent antimicrobial activity, indicating their potential for combating various infections [11]. In addition, micellar systems based on peptides exhibit excellent compatibility and biodegradability, a prolonged blood circulation time, non-toxicity, and non-immunogenicity [12]. An example of such a system is the ABD035 peptide with paclitaxel as a conjugated drug [4]. Enhanced stability is achieved as peptide amphiphiles self-assemble into micellar structures that remain stable under physiological conditions, which is crucial for preserving the bioactivity of the encapsulated peptides [13]. Micellar carriers enhance the controlled release of peptides by utilizing hydrophobic and electrostatic interactions between the peptide and the carrier. This controlled release is vital for maintaining therapeutic efficacy over time [14,15].

**Figure 4:**
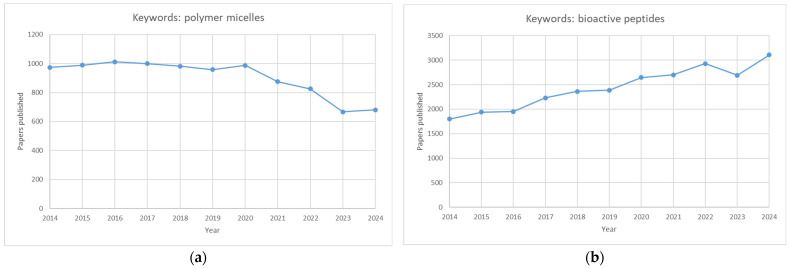
Graphs showing the number of publications over ten years (2014–2024). Graph (**a**) depicts the number of papers published using the keywords “polymer micelles”, whereas graph (**b**) depicts the number of papers published using the keywords “bioactive peptides”.

Although polymeric micelles and bioactive peptides have been extensively studied separately (Figure 4), micellar carriers of peptides need a more expansive scientific presentation, primarily because only around 10 papers were published on that issue in 2023.

## 2. Bioactive Peptides—Classification and Mode of Action

### 2.1. Classification of AMPs

AMPs are short peptides integral to the defense and innate immune systems of a variety of organisms, including bacteria, fungi, plants, fish, invertebrates, amphibians, crustaceans, insects, reptiles, mammals, and humans (see Table 1). They are categorized according to (1) source, (2) activity, (3) structural features, and (4) amino acid composition. The sources of AMPs include mammals (with human host defense peptides comprising a significant proportion), amphibians, insects, and microorganisms. Additionally, AMPs from marine environments have gained considerable interest.

**Figure 5:**
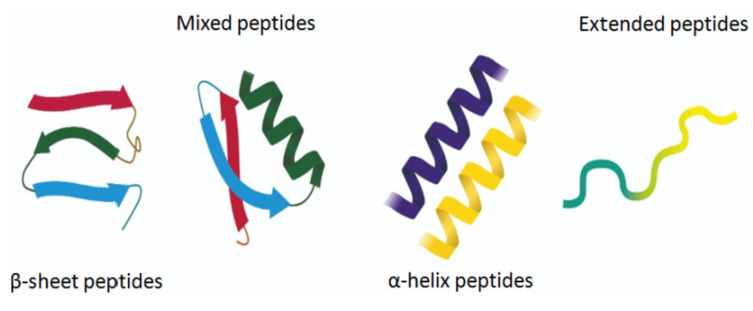
Different classes of AMPs [17].

The activities of AMPs can be divided into five primary categories: antibacterial, antiviral, antifungal, antiparasitic, and anti-tumor peptides. Furthermore, AMPs can be classified by their amino acid composition into categories such as proline-rich, tryptophan- and arginine-rich, histidine-rich, and glycine-rich peptides. Figure 5 illustrates different classes of AMPs [16,17].

The most widespread AMPs and their activity against given pathogens are summarized in Table 1.

**Table 1:** Sequences of some antimicrobial peptides, their sources, and their activity against pathogens.

	AMP	Source	Sequence	Active Against	Ref.
B A C T E R I A	Colicin M	*E. coli*	METLTVHAPSPSTNLPSYGNGAFSLSAPHVPGAGPLLVQVVYSFFQSPNMCLQALTQLEDYIKKHGASNPLTLQIISTNIGYFCNAERNLVLHPGISVYDAYHFAKPAPSQYDYRSMNMKQMSGNVTTPIVALAHYLWGNGAERSVNIANIGLKISPMKINQIKDIIKSGVVGTFPVSTKFTHATGDYNVITGAYLGNITLKTEGTLTISANGSWTYNGVVRSYDDKYDFNASTHRGVIGESLTRLGAMFSGKEYQILLPGEIHIKESGKR	*Enterobacter*, *Escherichia*, *Klebsiella*, *Morganella*, *Salmonella*, *Shigella*, and *Yersinia*	[18]
	Gramicidin	*B. brevis*	XGAXAXVXWXWXWXWX	Gram-positive bacteria; Gram-negative bacteria	[18]
	Microvionin	*Microbacterium arborescens*	MWQKAARGGSSVSPSEEFRRVLTLFATRSRASDRLHCRRDLGHGKEVELMSLEQLEALDASSEAAEMAASLGSQSC	Methicillin-resistant *S. aureus* (MRSA) and *Streptococcus pneumonia*	[18]
	Plantazolicin	*Bacillus amyloliquefaciens*	MTQIKVPTALIASVHGEGQHLFEPMAARCTCTTIISSSSTF	Closely related strains of genus *Bacillus*	[18]
	Sonorensin	*B. sonorensis*	MKGDIKMQPFHDEPQSLEMDQFQADDMTLWDAGRHHANVKHCASCWSCGSCASCWSCMGHSCWSCMGHSCWSCAGHSCWSCMGHSCWSCMGHSCWSCAGHCCGSCWHGGM	*B. subtilis*, *E. coli*, *Listeria monocytogenes*, *P. aeruginosa*, *S. aureus*, *Vibrio vulnificus*	[18]
	Nisin	*Lactococcus*, *Staphylococcu*, *Streptococcus* spp.	MSTKDFNLDLVSVSKKDSGASPRITSISLCTPGCKTGALMGCNMKTATCHCSIHVSK	Staphylococci, streptococci, enterococci, bacilli, listeria	[18]
	Epidermin	*S. epidermidis*	MEAVKEKNDLFNLDVKVNAKESNDSGAEPRIASKFICTPGCAKTGSFNSYCC	*S. hemolyticus*, *S. capitis*, *S. simulans*, *S. saprophyticus*, *S. hominis*, *S. epidermidis*, *S. aureus*	[18]
	Microcin L	*E. coli*	MKTWQVFFIILPISIIISLIVKQLNSSNLVQSVVSGIAIALMISIFFNRGK	*E. coli*, *S. enterica*, *Shigella* spp., *P. aeruginosa*	[18]
	Abp118	*Lactobacillus salivarius*	MKNLDKRFTIMTEDNLASVNGGKNGYGGSGNRWVHCGAGIVGGALIGAIGGPWSAVAGGISGGFTSCR	*Listeria monocytogenes*	[18]
	Pediocin	*Pediococcus* spp.	MKKIEKLTEKEMANIIGGKYYGNGVTCGKHSCSVDWGKATTCIINNGAMAWATGGHQGNHKC	*Listeria* spp.	[16]
P L A N T S	Mj-AMP2	Garden four-o’clock (*Mirabilis jalapa*)	MAKVPIAFLKFVIVLILFIAMSGMIEACIG NGGRCNENVGPPYCCSGFCLRQPNQG YGVCRNR	*B. megaterium*, *S. lutea*, *Magnaporthe oryzae*	[2]
	PmAMP1	Western white pine (*Pinus monticola*)	METKHLAYVMFVLVSLFLAMAQPSQA SYFSAWVGPGCNNHNARYNKCGCSNIS HNVHGGYEFVYQGQAPTAYNTNNCKG VAQTRFSSNVNQACSNFAWKSVFIQC	*Leptosphaeria maculans*	[2]
	SmAMP2	Chickweed (*Stellaria media*)	MLNMKSFALLMLFATLVGVTIAYDPNG KCGRQYGKCRAGQCCSQYGYCGSGSKY CAHNTPLSEIEPTAAGQCYRGRCSGGLC CSKYGYCGSGPAYCGLGMCQGSCLPDM PNHPAQIQARTEAAQAEAQAEAYNQA NEAAQVEAYYQAQTQAQPQVEPAVTK AP	*Alternaria* sp. and *Fusarium* sp.	[2]
	NpRS	Garlic (*Allium sativum*)	RSLNLLMFR	*C. albicans*	[2]
	SN1	Potato (*Solanum tuberosum*)	MKLFLLTLLLVTLVITPSLIQTTMAGSNF CDSKCKLRCSKAGLADRCLKYCGICCEE CKCVPSGTYGNKHECPCYRDKKNSKGKSKCP	*Rhizoctonia solani*	[2]
	SN2	Tomato (*Solanum lycopersicum*)	MAISKALFASLLLSLLLLEQVQSIQTDQVSSNAISEGADSYKKIDCGGACAARCRLSS RPRLCHRACGTCCARCNCVPPGTSGNTE TCPCYASLTTHGNKRKCP	*S. cerevisiae*	[2]
	CC-AMP1	Ghost Pepper (*Capsicum chinense* × *frutescens*)	ZETLDPICMAKCVLKCGKKAWCLTKCI AGCVL	*E. coli*, *K. pneumonia*, *A. baumannii*, *P. aeruginosa*	[2]
A N I M A L S	Cecropin A	Cecropia moth (*Hyalophora cecropia*)	KWKLFKKIEKVGQNIRDGIIKAGPAVAVVGQATQIAK-amide	*A. baumannii*, *P. aeruginosa*	[2]
	Melittin	Honey bee (*Apis mellifera*)	GIGAVLKVLTTGLPALISWIKRKRQQ-NH2	*S. aureus*, *A. baumannii*	[2]
	Piscidin 2	Schlegel’s black rockfish (*Sebastes schlegelii*)	MRFIMLFLVLSMVVLMAEPGEAFIHHIFG AIKRIFGDKQRDMADQQELDQRAFDRE RAFN	*S. aureus*, *Trypanosoma brucei*	[2]
	TBD-1	European pond turtle (*Emys orbicularis*)	YDLSKNCRLRGGICYIGKCPRRFFRSGSCS RGNVCCLRFG	*E. coli*, *L. monocytogenes*, MRSA, *C. albicans*	[2]
	Pelovaterin	Chinese soft-shelled turtle (*Pelodiscus sinensis*)	DDTPSSRCGSGGWGPCLPIVDLLCIVHV TVGCSGGFGCCRIG	*P. aeruginosa*, *Proteus vulgaris*	[2]
	Oh-Cath	King cobra (*Ophiophagus hannah*)	MEGFFWKTLLVVGALAIGGTSSLPHKP LTYEEAVDLAVSIYNSKSGEDSLYRLLE AVPPPEWDPLSESNQELNFTIKETVCLV AEERSLEECDFQEDGAIMGCTGYYFFGESPPVLVLTCKPVGEEEEQKQEEGNEEEKE VEKEEKEEDEKDQPRRVKRFKKFFKKLKNSVKKRAKKFFKKPRVIGVSIPF	*P. aeruginosa*, *Enterobacter aerogenes*	[2]
	Cancrin	Crab-eating frog (*Rana cancrivora*)	GSAQPYKQLHKVVNWDPYG	*E. coli*, *S. aureus*, *C. albicans*	[2]
	Magainin 2	African clawed frog (*Xenopus laevis*)	GIGKFLHSAKKFGKAFVGEIMNS	*E. coli*, *B. megaterium*, *A. baumannii*	[2]
	Buforin II	Asian toad (*Duttaphrynus melanostictus*)	TRSSRAGLQFPVGRVHRLLRK	*E. coli*, *S. typhimurium*, *S. aureus*, *B. subtilis*	[2]
	Indolicidin	Cattle (*Bos taurus*)	ILPWKWPWWPWRR-amide	HIV, *C. neoformans*, *C. albicans*, *Trichosporon beigelii*, *E. coli*, *P. aeruginosa*, *S. typhimurium*, *Staphyloccocus sp.*	[18]
	Protegrin	Pig (*Sus scrofa*)	RGGRLCYCRRRFCVCVGR-amide	*E. coli*, *S. aureus*, *P. aeruginosa*, *Chlamydia trachomatis*, *Neisseria gonorrhoeae*, *C. albicans*, HIV	[2]
	θ-defensin-1	Rhesus monkey (*Macaca mulatta*)	RCICTRGFCRCLCRRGVC	*S. aureus*, *C. albicans*	[2]
H U M A N	α-defensin 5, Paneth cell-specific	Human (*Homo sapiens*)	MRTIAILAAILLVALQAQAESLQERADEATTQKQSGEDNQDLAISFAGNGLSALRTSGSQARATCYCRTG RCATRESLSGVCEISGRLYRLCCR	*S. aureus*, *E. coli*	[2]
	LL-37	Human (*Homo sapiens*)	LLGDFFRKSKEKIGKEFKRIVQRIKDFLR NLVPRTES	Gram-positive; Gram-negative	[2]
	HNP-1	Human (*Homo sapiens*)	ACYCRIPACIAGERRYGTCIpYQGRLWA FCC	*E. coli*, *S. aureus*, *S. epidermis*	[2]
	KYE28	Human (*Homo sapiens*)	KYEITTIHNLFRKLTHRLFRRNFGYTLR	*E. coli*, *P. aeruginosa*, *B. subtilis*, *S. aureus*, *C. albicans*	[18]
	HBD-2	Human (*Homo sapiens*)	GIGDPVTCLKSGAICHPVFCPRRYKQIGT CGLPGTKCCKKP	*P. aeruginosa*, *E. coli*, *C. albicans*	[2]

**Figure 6:**
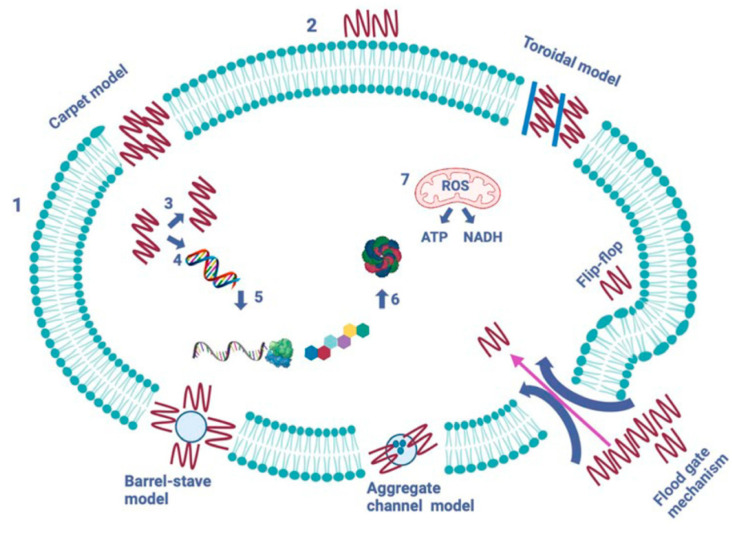
Modes of action of antimicrobial peptides [2]. The image depicts two primary types of peptides: membrane-bound and intracellular active peptides. The cytoplasmic membrane of a pathogen cell is represented by the outer layer, labeled “1”, while the AMPs are indicated by “2”. Membrane-bound peptides operate through five mechanisms: barrel-stave, toroidal, carpet, floodgate, and aggregate channel models. Intracellular AMPs, marked with numbers “3” to “7”, demonstrate the inhibition of enzymes necessary for binding cell wall structural proteins, as well as for DNA and RNA synthesis, ribosomal activity, chaperone protein synthesis, and cellular respiration, which involves the formation of reactive oxygen species (ROS) and the usage of ATP (adenosine triphosphate) and NADH (nicotinamide adenine dinucleotide).

AMPs operate through five mechanisms to create pores and channels in the pathogen’s cytoplasmic membrane (see Figure 6) [19,20,21]. The formation of pores induced by peptides aims to facilitate the leakage of cytoplasmic contents across the lipid bilayer. The barrel-stave model, established in 1977, describes how peptides form transmembrane pores by targeting the lipid core of the cytoplasmic membrane. In contrast, the toroidal model, introduced in 2001, represents a cascading accumulation of peptides that continuously puncture the cell membrane. These aggregated peptides promote the targeting and binding of additional peptides to the lipid monolayer, resulting in more membrane pores. The carpet model, proposed in 1992, illustrates AMPs acting like detergents, solubilizing and destabilizing the cytoplasmic membrane, which takes on a carpet-like structure. The aggregate channel model, put forth in 2012, suggests that AMPs encircle the membrane as spontaneously formed peptide aggregates, forming channels that enable the escape of cytoplasmic contents, including all organelles. Lastly, in the floodgate mechanism (proposed in 2011), α-helical AMPs create temporary toroidal openings in the cell membrane at the onset of their action. This occurs as these peptides draw in nearby unbound peptides, further aiding in creating additional holes in the cytoplasmic membrane [22].

### 2.2. Anticancer Peptides

Anticancer peptides (ACPs) have emerged as an effective cancer treatment due to their high specificity and lower toxicity [23,24]. They specifically target cancer cells at various stages: initiation, promotion, and progression. ACPs offer advantages over chemotherapeutic agents, including greater specificity, enhanced tumor penetration, reduced side effects, and more straightforward modifications.

Differences in cell membrane properties enhance ACP specificity between cancerous and healthy cells. Anionic molecules, such as phospholipids and sialic acid, impart a negative charge to cancer membranes, facilitating electrostatic interactions with ACPs. Greater fluidity in cancer cells enables destabilization and lysis by ACPs. They penetrate cells directly or via endocytosis, causing cytotoxic effects. Melittin and ZXR-2 induce membrane lysis, whereas ChMAP-28 and LFchimera initiate necrosis. Some disrupt mitochondrial integrity, causing intrinsic apoptosis (e.g., ZXR-1 and bovine lactoferrin peptides). Others activate extrinsic apoptosis by binding to death receptors, such as Melittin and anti-DR5 peptides, and some induce both apoptosis pathways, like HPRP-A1 and DN1 [23,25].

ACPs can damage cancer DNA directly or indirectly through various mechanisms depending on the cancer type and stage. They may interact with DNA to induce strand breaks or cross-linking, causing cell death (e.g., TH2–3 and HNPs). Some ACPs inhibit DNA synthesis by binding to specific enzymes, while others, such as Lunasin, target DNA-associated proteins, including histones, thereby altering gene expression [23,26].

Anti-angiogenic peptides (AAPs) interact with components of the angiogenic process, including growth factors, enzymes, and receptors, which are crucial for cancer cells’ blood vessel formation. Key targets include platelet-derived growth factor, vascular endothelial growth factor (VEGF), and angiopoietins. Peptides such as AC-P19M, Cilengitide, IM862, and TM inhibit new blood vessel formation by blocking related signaling pathways [23].

ACPs target cell growth proteins, halting the cell cycle and inhibiting cancer cell proliferation. They modulate cyclin and CDK activity. Most ACPs inhibit proliferation by arresting cells at the G1 phase and inducing apoptosis. For example, Gonearrestide, a novel ACP derived from scorpion, inhibits growth by inducing G1 phase arrest via CDK4 inhibition, while rapeseed peptide induces G0/G1 phase arrest through the P53 pathway.

Migration, invasion, and metastasis involve extracellular matrix degradation and epithelial–mesenchymal transition (EMT). ACPs inhibit cancer cells’ migratory, invasive, and metastatic abilities by regulating pathways such as the PI3K/AKT/mTOR and Wnt pathways. Peptides like Foxy-5, Q7, AC-P19M, TH2–3, and SMP24 effectively suppress these processes.

ACPs promote cancer cell differentiation, reducing their proliferation and migration. These differentiated cells are less aggressive and more responsive to treatment, providing a strategic approach to cancer therapy. Examples include Bryostatin 1 and D-pep-P6, which activate protein kinase C and the TLR-2 signaling pathway to induce the differentiation of leukemic cells.

ACPs reverse drug resistance by altering cellular processes that enable cancer cells to survive chemotherapy. Key resistance mechanisms of cancer cells include drug efflux, inactivation, target modification, and epigenetics. ACPs block transporter proteins, causing drug efflux, such as HX-12 C peptide, which inhibits ABCB1 (ATP Binding Cassette Subfamily B Member 1) function. They also enhance drug uptake using GRR10W4 (GRRPRPRPRPWWWW-NH_2_) and PNC27–Doxil (ACP-chemotherapy drug). The sequence of PNC27 is PPLSQETFSDLWKLLKKWKMRRNQFWVKVQRG.

ACPs influence multiple immune cells, including tumor-associated macrophages (TAMs), regulatory T cells (Tregs), T cells, natural killer (NK) cells, and dendritic cells (DCs), by reversing the suppressive tumor microenvironment to an antitumor one, making them promising tools for tumor immunotherapy. The key mechanisms of ACPs include (i) altering cytokine profiles (e.g., CSSTRESAC and silk peptide), (ii) reprogramming M2-like TAMs (e.g., TAMpepK and RP-182), (iii) activating CD8+ T cells (e.g., cyclic peptide C25), (iv) reducing CD4+ Treg cells (e.g., cyclic peptide C25), (v) activating NK cells (e.g., Mel-P15), and (vi) disrupting protein–ligand interactions (e.g., CLP002 and p344).

Cell-penetrating peptides (CPPs) transport molecules across membranes, serving as carriers for tumor-targeting agents [21,25]. For example, the ANTP-SmacN7 fusion peptide uses ANTP to deliver SmacN7 into cells for radiosensitization. Tumor-homing peptides (THPs) enhance drug accumulation at tumors or deliver therapeutic proteins, such as peptides 22, HN-1, and KLA-RGD. Tumor-specific monoclonal antibodies improve the efficacy of ACPs in cancer treatment. Monomethyl auristatin E (MMAE) and F (MMAF) disrupt tubulin polymerization in the cancer cell, exhibiting 100- to 1000-fold stronger cytotoxicity than doxorubicin. Six ADCs, including Adcetris and Polivy, are FDA-approved with these payloads; Telisotuzumab and Zilovertamab are currently in Phase III trials.

P-PROTACs (proteolysis-targeting chimeras) link ACPs to target specific cancer cell proteins with E3 ligase inhibitors for degradation. They offer easy design, high specificity for hard-to-drug proteins, resistance to mutations, and low toxicity, making them promising for cancer therapy. Examples include (i) FOXM1-PROTAC, which degrades FOXM1 (Forkhead box protein M1), reducing GLUT1 (Glucose transporter 1) and PD-L1 (Programmed death-ligand 1) expression and resulting in cancer suppression; (ii) AR pep-PROTAC, which lowers AR (androgen receptor) levels and induces tumor regression; and (iii) xStAx-VHLL, which promotes human β-catenin degradation and hinders Wnt signaling pathway-dependent tumor growth [23]. Examples of ACP sequences are presented in Table 2.

**Table 2:** Sequences of some anticancer peptides, their sources, and their activity.

ACPs	Source	Sequence	Ref.
ZXR-2	Fattail scorpion (*Androctonus mauritanicus*)	MNKKTLLVIFFITMLIVDEVNSFKIGGFIKKLWRSKLAKKLRAKGRELLKDYANRVINGGP EEEAAVPAERRR	[18]
TAT	HIV	GRKKRRQRRRPQ	[18]
AP	synthetic construct	CRKRLDRN	[18]
Lactoferrin, partial	Domestic cattle (*Bos taurus*)	YTRVVWXAVGPEEQKKXQ	[18]
ChMAP-28	Domestic goat (*Capra hircus*)	GRFKRFRKKLKRLWHKVGPFVGPILHY	[18]
LFchimera	synthetic construct	MDLIRKLLSKAQEKFGKNKSRKGLKKMRWQWRRCKFHHHHHHKDEL	[18]
TH2-3	synthetic construct	QSHLSLCRWCCNCCRSNKGC-NH2	[18]
HX-12C	synthetic construct	FFRKVLKLIRKIWR	[18]

### 2.3. Anti-Inflammatory Peptides

Numerous natural peptides exhibit either anti-inflammatory or pro-inflammatory properties [27]. Notably, many antimicrobial peptides possess anti-inflammatory characteristics, effectively targeting Gram-negative and Gram-positive bacteria, *Mycobacterium tuberculosis*, fungi, and cancerous cells. Currently, significant interest centers on peptides that can replicate the functions of mediators implicated in inflammation-related diseases. While some peptides are found naturally in their sources, most are integrated within the structure of related proteins and can be released through digestion or be designed based on their structure. Various proteins and peptides derived from eggs, milk, soy, plants, and marine sources have shown anti-inflammatory effects [28].

Various peptide-based mimetics of SOCSs (suppressors of cytokine signaling) are involved in neonatal fatal inflammatory diseases, autoimmune encephalitis, and inflammation-related cancer processes. Additionally, a small inhibitor of Aminopeptidase N-terminal is noted in neuroendocrine prostate cancer. A peptide derived from Chromofungin can suppress macrophage pro-inflammatory actions by disrupting TLR4/NF-kB signaling and inhibiting NF-κB activation. Furthermore, peptides relevant in autoimmune diseases include [K6T] P8, which inhibits the IL-15 receptor, and Cyclotide [T20K] kalata B1, known for blocking the proliferation of immune-competent cells in multiple sclerosis.

Among the peptides involved in neurological diseases, a segment of the NCAM protein known as FGL peptide stimulates IL-4 secretion from microglial cells, increasing CD200 and reversing age-related hippocampal decline in vivo. Additionally, the MHP1 peptide exhibits anti-osteoclast activity in cases of ischemic stroke by inhibiting LPS and TNF-α production. The examples provided suggest that the development of peptide-based drugs will continue to expand, but enhancing their effectiveness through chemical optimization is essential. Numerous strategies can be used in tandem to modify peptides for improved therapeutic outcomes. One method involves introducing conformational constraints, such as mono- or bi-cyclic structures, to reduce the conformational flexibility of linear peptides. Such changes often lead to better membrane permeability and increased resistance to proteolysis by endo- and exopeptidases. Another strategy includes replacing natural amino acids with unnatural ones and/or N-methyl-α-amino acids, which also boosts the plasma stability of the compound. Moreover, emerging peptide technologies—including multifunctional peptides, cell-penetrating peptides, and peptide–drug conjugates—will enhance the potential of peptides as treatments for inflammatory disorders and beyond [28]. Table 3 illustrates the sources, activities, and sequences of some anti-inflammatory peptides (AIPs).

**Table 3:** Sequences of some anti-inflammatory peptides, their sources, and their activity.

AIP	Source	Sequence	Ref.
Kalata B1	Synthetic construct	AGETCVGGTCNTPGATCSWPVCTRNGLPV	[18]
FGL	Human (*Homo sapiens*)	EVYVVAENQQGKSKA	[18]
MHP1	Synthetic construct	LMVYVVKTSIKIPSSHNLMKGGSTKNWSGN	[18]
Casein	Buffalo milk	YQEPVLGPVR	[29]
Cliotide T28	Asian pigeonwings (*Clitoria ternatea* L.)	GGSIPCGESCVFLPCFLPGCSCKSSVCYLN	[29]
Labatidin	Coral plant (*Jatropha multifida* L.)	AGVWTVWGTI	[29]
LR13	Rice (*Oryza sativa* L.)	LLPPFHQASSLLR	[29]

### 2.4. Immuno-Modulatory Peptides

Peptides and peptidomimetics serve as immune-modulators by inhibiting or enhancing the immune response to promote tolerance. Understanding B cell and T cell epitopes, alongside conformational constraints, is crucial for designing peptide-based immune-modulating agents. Advances in peptide conformation, synthesis, and modified amino acid side chains have created new therapeutic options for autoimmune diseases and cancer. In cancer treatment, peptide epitopes help train the immune system to identify and combat cancer cells locally and systemically [30].

Peptides mimic the surfaces of specific proteins, disrupting protein–protein interactions (PPIs) and modulating signaling pathways. This modulation is crucial in immune responses as these molecules do not completely inhibit signaling but regulate it. Peptide-based medications offer benefits such as high specificity, low immunogenicity, and the ability for large-scale synthesis. Most peptides lack tertiary and quaternary structures, which enhances their stability compared to antibodies. They effectively merge the advantageous characteristics of small-molecule drugs and protein therapeutics. The sources, activity, and sequences of selected plant-derived immuno-modulatory (IMP) peptides are presented in Table 4.

**Table 4:** Sequences of some immune-modulatory peptides, their sources, and their activity.

IMP	Source	Sequence	Ref.
PEP1	Rice (*Oryza sativa* L.)	GIAASPFLQSAAFQLR	[18]
cliotide T32	Asian pigeonwings (*Clitoria ternatea*)	GDLFKCGETCFGGTCYTPGCSCDYPICKNN	[18]
TK17	Rice (*Oryza sativa* L.)	TPMGGFLGALSSLSATK	[29]
cycloviolacin O2	Common violet (*Viola odorata* L.)	GIPCGESCVWIPCISSAIGCSCKSKVCYRN	[29]
Viphi A	Chinese violet (*Viola philippica*)	GSIPCGESCVFIPCISSVIGCACKSKVCYKN	[29]
Limyin	Lima bean (*Phaseolus limensis*)	KTCENLATYYRGPCF	[29]

### 2.5. Regenerative Peptides

Numerous potential therapies are currently in animal trials, concentrating on delivering growth factors linked to soft tissue repair. Whether aimed at recovering cartilage, the extracellular matrix (ECM), muscle, tendons, ligaments, or aiding in bone repair, advancements in peptide therapy present promising avenues for joint regeneration and for preventing future degeneration. Collagen-2, hydrolyzed collagen (HC), bepecin, the human peptide GHK (glycyl-l-histidyl-l-lysine), and thymosin β4 have various applications in medicine and biotechnology, offering multiple opportunities for research and clinical applications. Peptide therapeutics represent a promising aspect of regenerative medicine, aiming to provide a minimally invasive approach for managing soft tissue degeneration [31].

### 2.6. Dermal Peptides

Signal peptides are biologically active compounds that can mitigate skin aging by stimulating fibroblast activity. This stimulation produces heightened biological responses, including increased collagen production, elastin, fibronectin, glycosaminoglycans, and proteoglycans [32]. Additionally, these peptides function as growth factors by activating protein kinase C, which is crucial for cell growth and migration. Signal peptides boost fibroblast metabolism or accelerate hair growth [33].

### 2.7. Anti-Diabetic Peptides

Anti-diabetic peptides primarily inhibit the activity of dipeptidyl peptidase-IV (DPP-IV). Recent studies highlighting hypoglycemic mechanisms have identified five distinct inhibitors: (a) α-glucosidase inhibitors, (b) α-amylase inhibitors, (c) dipeptidyl peptidase-IV inhibitors, (d) inhibitors of the glucose transporter system, and (e) insulin mimetics [34]. A wide range of regenerative, dermal, and anti-diabetic peptides is available, with some listed in Table 5.

**Table 5:** Sequences of some regenerative, dermal, and anti-diabetic peptides, their sources, and their activity.

	AMP	Source	Sequence	Ref.
Regenerative	GHK	Human plasma, saliva, and urine	GHK	[18]
	Thymosin β4	Human (*Homo sapiens*)	MSDKPDMAEIEKFDKSKLKKTETQEKNPLPSKETIEQEKQAGES	[18]
	Bepecin	Synthetic	GEPPPGKPADDAGLV	[18]
Dermal	Lipospondin	Synthetic	Elaidyl-KFK-OH	[35]
	GEKG	Synthetic	GEKG	[35]
	PKEK	Synthetic	PKEK	[35]
Anti-diabetic	CSP4	Cumin seed (*Cuminum cyminum*)	RCMAFLLSDGAAAAQQLLPQYW	[36]
	KLPGF	Albumin	KLPGF	[36]
	Vglycin	Pea seeds (*Pisum sativum*)	VSCNGVCSPFEMPPCGSSACRCIPYGLVVGNCRHPSG	[36]

## 3. Polymer Micelles—Types, Properties, and Methods for Characterization

### 3.1. Types of Polymer Micelles

Block and graft copolymers with amphiphilic character, having a large solubility difference between hydrophilic and hydrophobic segments, tend to aggregate in an aqueous medium into PMs of various types and dimensions ranging from 10 to 100 nm [37]. PMs are formed when the copolymer concentration exceeds the critical aggregation concentration (CAC) (also known as the critical micelle concentration (CMC)). Some temperature-responsive copolymers aggregate above the so-called critical micelle temperature (CMT). Above the CMC (and CMT), a predominant intermolecular interaction occurs between the hydrophobic segments of copolymers, usually forming multimolecular micellar structures [38]. The CMC and CMT of copolymers are essential for the self-assembly and thermodynamic stability of PMs in solution. Also, PMs can be formed by electrostatic interactions of oppositely charged macrochains (polyion complex micelles). Depending on the copolymer composition and interchain interactions, various morphologies of micelles can be designed—spherical, worm-like, disk-like, rod-like, multicompartment, etc. [39]. In general, these micelles may consist of a hydrophobic core and hydrophilic shell, whereas in a reverse micelle (formed in organic solvent), this arrangement can be switched to a hydrophilic core and a hydrophobic shell [40]. More complex micellar structures are fabricated by blending two or more copolymers of different composition (mixed micelles).

Polymeric micelles can be constructed using biocompatible synthetic polymers and natural polymers, which can impart the processability and adaptability of the former and the ability of the latter to program assemblage mechanisms and control the structure and function [41]. Regarding their surface charge, PMs are nonionic, anionic, and cationic. PMs can be additionally stabilized by physical or chemical crosslinking to avoid spontaneous disaggregation. Based on the mode of encapsulation of drugs, PMs are mainly classified into two types—micelles with covalently bonded drugs and micelles with physically entrapped drugs [42]. The drugs can be embedded in different regions of PMs depending on the polarity and/or specific molecular interactions.

The nature of building polymer segments determines the behavior and performance of PMs [43,44]. Thus, polymeric micelles are designed to respond to stimuli such as pH, temperature, light, enzymes, redox conditions, etc. Such stimuli-responsive micelles are advantageous for controlled and triggered drug release in a specific zone/organ in response to pathological conditions or external stimuli [45].

**Figure 7:**
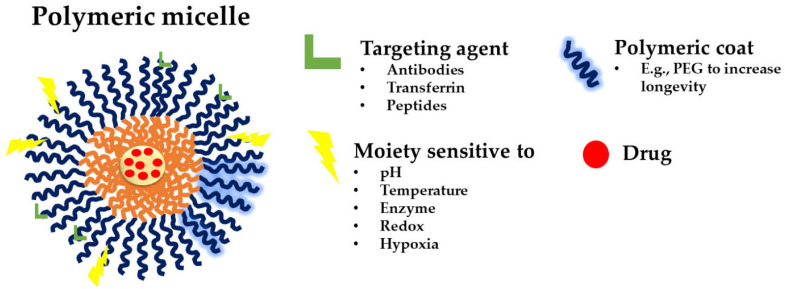
A schematic representation of a multifunctional polymeric micelle [47]. The figure depicts possible targeting agents, such as antibodies, transferrin, and peptides (green); the polymeric coating (blue; PEG given as an example); and the different moieties sensitive to pH, temperature, enzymes, redox, and hypoxia (yellow); and the loaded drug (red).

Until now, three generations of micellar delivery systems, i.e., polymeric micelles for passive, active, and multifunctional drug targeting, have been developed, with each subsequent generation displaying greater specificity for the diseased tissue and/or targeting efficiency [46]. Micelles for active targeting are engineered by modifying the micellar shell with targeting ligands for specific cell or tissue accumulation. They are predominantly used in cancer therapy to improve drug accumulation at the tumor site and reduce off-target effects. Peptides can be used to functionalize micellar carriers, enhancing their targeting capabilities. For instance, cell-penetrating peptides (CPPs) and cell-targeting peptides (CTPs) are frequently employed to improve cellular uptake and targeting specificity [14]. Theranostic micelles combine therapeutic and diagnostic functions. They are used for simultaneous drug delivery and imaging, making them suitable for personalized medicine and real-time monitoring of treatment efficacy. PMs that integrate several functions into a single carrier are named multifunctional polymeric micelles (Figure 7) [47].

**Figure 8:**
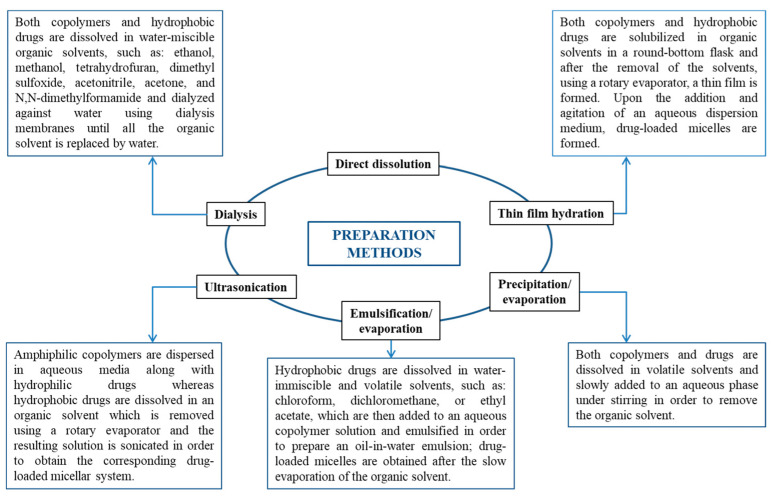
Preparation methods of both empty and drug-loaded micellar systems [4].

Depending on the development method, micelles can be created using various techniques, such as direct dissolution, dialysis, solvent evaporation, film hydration, etc. (Figure 8) [4].

#### 3.1.1. Natural Polymer Micelles

Natural polymers are generally incapable of self-assembly; creating micelles necessitates chemical alteration of biopolymer molecules to introduce hydrophobic functional groups. Once enabling self-assembly, natural polymer micelles offer significant advantages for controlled release, targeted drug delivery, and solubilizing hydrophobic active materials, all while ensuring intrinsic biocompatibility and biodegradability—an inherent concern with synthetic polymers [48]. Natural polymeric micelles based on chitosan, alginate, and collagen have recently garnered substantial attention as drug delivery systems in pharmaceuticals. For instance, uronic-rich polysaccharides like alginate are altered through carboxylic group amidation or esterification, while aminated polysaccharides undergo modifications such as amine function quaternization, N-acylation, and N-alkylation.

#### 3.1.2. Synthetic Polymer Micelles

Various biocompatible and biodegradable synthetic polymers are employed in micelle preparation for biomedical applications. Some of the widely accepted hydrophobic (co)polymers that are used in PMs are poly(caprolactone) (PCL), poly(lactide) (PLA), poly(lactide-co-glycolide) (PLGA), poly(propylene oxide), and poly(amino acids) [49]. PMs comprising biodegradable polymers or cleavable bonds have a low toxicity risk as they break down into single polymer chains or even smaller fragments that can be easily eliminated without causing toxicity [50]. Poly(ethylene glycol) (PEG), also known as poly(ethylene oxide) (PEO), is the most commonly used hydrophilic polymer for developing micellar nanocarriers. The brush conformation of PEG macromolecules leads to a “stealth” behavior of the nanocarriers with inhibited uptake by phagocytic cells [51]. Other suitable non-ionic hydrophilic polymers are poly(N-(2-hydroxypropyl)methacrylamide)(PHPMA), polyglycidol (PG), poly(2-methyl-2-oxazoline) (PMeOx), poly(2-ethyl-2-oxazoline) (PEtOx); poly(vinyl alcohol)(PVA), polyacrylamide, polyvinylpyrrolidone (PVP), etc. Polyanions such as poly(acrylic acid) and poly(methacrylic acid) are used for their bioadhesive properties and complexation with peptides. Synthetic polycations, including polyethylenimine (PEI), poly(2-(dimethylamino)ethyl methacrylate) (PDMAEMA), and polylysine, are useful delivery vehicles for nucleic acids and proteins.

### 3.2. Key Properties of Polymer Micelles

Micellar carriers are increasingly used to deliver peptides due to their unique properties and advantages. The key characteristics that make micellar carriers effective for peptide delivery involve their specific structure, small size, narrow particle size distribution, versatility and modifiability, high colloidal stability both in vitro and in vivo, ease of preparation, and low toxicity (Table 6). Such a diverse set of characteristics makes PMs advantageous for delivering BPs compared to other nanocarriers (e.g., liposomes, nanogels, or nanoparticles). PMs, comprising a hydrophobic core and a hydrophilic shell, can encapsulate peptides, protecting them from enzymatic degradation and other destabilizing factors in the biological environment and enhancing their stability and bioavailability [52,53,54,55,56]. Cross-linking micelles can improve their stability against environmental changes such as dilution and ionic strength, which is crucial for maintaining the integrity of the peptide cargo [57]. The hydrophobic and hydrophilic segments can be chemically tuned to optimize peptide immobilization through non-covalent interactions (e.g., electrostatic or hydrophobic) or covalent conjugation, maintaining the peptides’ biological activity [58]. Micelles can be designed to respond to specific stimuli such as pH, temperature, or enzymatic activity, allowing for controlled and targeted release of peptides [53,58,59]. Micelles can be engineered to target specific tissues or cells, reducing off-target effects and improving therapeutic efficacy. This is achieved by incorporating targeting ligands or exploiting the enhanced permeability and retention (EPR) effect in tumors [59,60]. The preparation of micellar carriers is relatively straightforward and can be scaled up for industrial production, facilitating their transition from research to clinical application [57]. Micelles are often made from biocompatible and biodegradable materials, such as PEGylated phospholipids, which are safe for human use and have been approved in marketed products [57].

**Table 6:** Summary of micellar carriers’ properties.

Property	Description	References
Amphiphilic Nature	Self-assembly into a core–shell structure with a hydrophobic core and a hydrophilic shell	[52,58]
Chemical Versatility	Tunable segments for peptide immobilization and maintaining biological activity	[58]
Stimuli-Responsiveness	Controlled release in response to specific stimuli	[53,58,59]
Protection from Degradation	Protects peptides from enzymatic degradation and environmental factors	[54,55,56]
Cross-Linking for Stability	Enhances stability against environmental changes	[57]
Improved Solubility	Enhances the solubility and bioavailability of peptides	[52]
Targeted Delivery	Engineered for specific tissue or cell targeting	[59,60]
Simple Preparation	Easy and scalable preparation methods	[56]
Low toxicity	Made from safe, biocompatible, and biodegradable materials	[56]

These properties collectively make micellar carriers a promising and versatile platform for effectively delivering peptide therapeutics. An “optimal” nanocarrier should possess the capacity to effectively solubilize a variety of poorly soluble pharmaceutical agents, biocompatibility, longevity, high stability in vitro and in vivo, and the ability to accumulate in pathological areas with compromised vasculature. Table 7 lists the main benefits of PMs with respect to their use as carriers of BPs.

**Table 7:** Summary of advantages of using micellar carriers for peptides.

Aspect	Details	References
Biocompatibility	Good compatibility, biodegradability, and non-toxicity	[4]
Stability	Stable under physiological conditions, controlled release	[12,13]
Functionalization	Surface modification with CPPs and CTPs for targeting	[14]
Applications	Targeted drug delivery, cancer therapeutics, and diagnostic applications	[7,12]
Challenges	Environmental sensitivity and controlled release mechanisms	[57]

### 3.3. Methods for Characterization of Polymer Micelles

The physicochemical properties of PMs influence their interactions with cells; therefore, evaluating the developed nanocarriers is an essential step before practical use. When possible, more than one technique should be employed to assess the given characteristics of nanomaterials. There are microscopy-based techniques (e.g., SEM, HR-TEM, and AFM—the full names of the techniques are presented in Table 8), which provide information on the size and morphology of PMs. Other techniques, based on the light scattering phenomena, are used to determine the particle size and size distribution, aggregation number, etc. Examples of these techniques are DLS and SLS. Many other methods provide further information on the structure, composition, optical properties, and other common and more specific physical properties of PMs. Examples of these techniques include small-angle X-ray, spectroscopy, and neutron scattering techniques (see Table 8).

## 4. Applications of Polymer Micelles as Carriers of Bioactive Peptides

**Figure 9:**
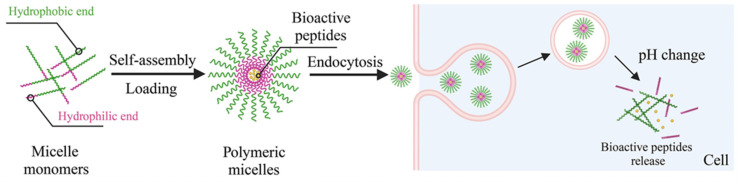
Loading PMs with BPs via self-assembly and the mechanism of delivery [66].

The encapsulation of peptides within polymeric micelles helps preserve the structural integrity of AMPs, ensuring they maintain their plasma membrane- and cell wall-disrupting capabilities [61,62]. There are three primary peptide-loading methods for polymeric micelles: physical encapsulation by co-assembly, electrostatic interaction, and chemical bonding. BPs are mixed with copolymeric materials in the physical co-assembly method, allowing them to spontaneously encapsulate into the micellar core during self-assembly without needing specific functional groups for chemical bonding. Electrostatic interaction is one type of physical method that uses electrostatic forces to firmly attach charged peptides to the polymeric micelles, occurring during micelle formation. This method is particularly effective under mild conditions. Conversely, the chemical method involves covalently linking peptide molecules to reactive groups at the hydrophobic sites of the block copolymer, necessitating specific chemical reactions [58,59]. Reactive groups on the block copolymer are required to enable this covalent interaction with the loaded BPs. Overall, PM encapsulation provides notable benefits for BP delivery, such as selective targeting of specific body sites, reduced systemic degradation, and increased bioavailability [63]. Micellar carriers promote more significant BP accumulation at the therapeutic site, enhancing efficacy. For instance, double-modified starch micelles, made with modified sulfobetaine and deoxycholic acid, effectively encapsulated walnut peptides, improving stability, enhancing antioxidant activity, and controlling release. Additionally, polymeric micelles excel at delivering poorly water-soluble peptides due to the hydrophobic nature of their core. Improving solubility and stability significantly boosts the bioavailability of these BPs, which is one of their most important benefits [64]. The micellar carriers aid the delivery of AMPs to the intracellular environment, where they can exert these additional antibacterial effects (Figure 9) [65]. Combining AMPs with other antimicrobial agents within polymeric micelles can yield synergistic effects, enhancing overall antimicrobial activity [66].

### 4.1. Antibacterial and Antifungal Activity

Thermo-sensitive polymeric micelles based on a poly(2-(adenine-9-yl) ethanol methacrylate-cosulfobetaine methacrylate) (poly(AEM-co-SBMA) copolymer enhanced the antibacterial activity of alamethicin against Staphylococcus aureus. It was suggested that the assembly complex showed higher antibacterial activity than the free alamethicin due to enhanced enzyme stability, originating from the protection effect of PMs [67].

Polymer–peptide systems comprising GKWMKLLKKILK-NH_2_ oligopeptide, covalently bound via various biodegradable spacers to a hydrophilic biocompatible polymer carrier, sensitive to a change in pH, demonstrated high antibacterial efficacy against *S. epidermidis*, *E. coli*, and *A. baumannii* [65]. In this case, it was necessary to release the peptide from the polymer carrier in response to a pH decrease.

Zhang et al. developed a novel AMP amphiphilic conjugate, DP7-C, by modifying a previously discovered highly active AMP with cholesterol. DP7-C spontaneously self-assembled into stable nanosized micelles in an aqueous medium. The DP7-C micelles showed lower hemolytic activity than their unconjugated counterparts toward human red blood cells. An investigation into the molecular mechanism further suggested that DP7-C could regulate immune responses with its direct antibacterial activities [68].

Machado et al. [69] developed a self-assembled micelle carrier for AMP LL18 using dextrin functionalized with succinylated vitamin D3 and succinic anhydride. This amphiphilic material self-assembled into micelles in aqueous media and efficiently loaded LL18 through electrostatic and hydrophobic interactions. This formulation significantly improved the antibacterial activity of LL18 against *S. aureus* and reduced its cytotoxicity. The system is a promising platform for developing multifunctional antibiotic-independent antimicrobial agents not prone to developing bacterial resistance to treat bone infections.

In a study by Kim et al. [70] (HKK)2 (histidine-lysine-lysine) and (HKK)6, two antifungal peptides with “HKK” repeated motifs, were covalently bound with PEGylated 1,2-distearoyl-sn-glycero-3-phosphoethanolamine (DSPE) and formulated to encapsulate AmB with methoxy poly(ethylene glycol)-b-PLGA (mPEG-PLGA).

Polymeric micelles can significantly increase the solubility of poorly soluble antifungal agents like amphotericin B (AmB) and itraconazole (ICZ), leading to improved therapeutic efficacy [71,72,73,74,75,76]. Voriconazole has also been incorporated into micelles, resulting in highly effective delivery through inhalation [77]. The targeted delivery of miconazole (MCZ) employing LL37 Fragment Mutant Peptide CKR12-poly(lactic acid-co-glycolic acid) micelles showed high antifungal activity, emphasizing that the micelles disrupted both the cell wall and the cell membrane of *C. albicans* [66]. Thus, the attachment of AMPs to the surface of CKR12-PLGA-MCZ micelles seems to be a promising approach for microbial targeting and maximizing the performance of MCZ.

### 4.2. Antiviral Activity

Polymeric micelles have shown promising results in preclinical studies for antiviral peptides, demonstrating improved delivery, stability, and therapeutic outcomes [78,79]. These micelles have been used to deliver peptides that inhibit HIV entry by targeting viral envelope proteins, showcasing improved pharmacokinetics and therapeutic efficacy [80,81].

Conjugating PEG chains to an anti-HIV peptide FI resulted in micellar complexes with prolonged half-lives and potent antiretroviral activity against 47 HIV-1 [82]. The plasma half-life of PEGylated peptide was up to 4.6-fold longer (5.1 h) than that of the non-modified peptide (~1.1 h).

Giacon et al. [83] developed PLGA-PEG-Bis-sulfone nanoparticles (PPB-NPs) externally functionalized with ZO-1’s PDZ2 domain as a possible adjuvant therapeutic strategy for inhibiting or retarding SARS-CoV-2-mediated pathogenesis. These results seem promising for developing targeted adjuvant antiviral therapies that hinder virus entry and replication and effectively attenuate the associated virulence factors, potentially revolutionizing our ability to combat viral infections [83].

### 4.3. Antitumor Activity

Typically, peptides are conjugated to polymeric micelles to enhance the targeting and therapeutic efficacy of antitumor drugs [84,85]. For example, the Flt1 peptide conjugated with hyaluronate (HA) in micelles encapsulating epirubicin has shown promising results in the treatment of hepatocellular carcinoma. [86]. Peptides like TAT and AP enhance the internalization of micelles into tumor cells via receptor-mediated endocytosis, improving drug delivery efficiency [87]. PMs aimed at active tumor targeting and tumoral pH responsiveness were synthesized by combining the AP peptide (CRKRLDRN) conjugated PEG–poly(d,l-lactic acid) block copolymer (AP–PEG–PLA) with pH-sensitive micelles consisting of methyl ether poly(ethylene glycol) (MPEG) and poly(β-amino ester) (PAE) block copolymers (MPEG-PAE). After loading with the anticancer drug doxorubicin (DOX), the system exhibited excellent anticancer therapeutic efficacy compared to free DOX and DOX-encapsulated MEG-PAE micelles [88].

A Cyclic-Arg-Gly-Asp (cRGD) peptide attached to an acetal-poly(ethylene glycol)-b-poly(β-benzyl L-aspartate) (Ac-PEG-b-PBLA) copolymer was combined with MeO-PEG-PBLA-Ac at a ratio of 1:3 to create epirubicin-loaded cRGD micelles (cRGD-Epi/m). These cRGD-Epi/m were effective against an orthotopic GBM model, achieving enhanced accumulation and penetration in the tumors. [89].

Wang et al. developed a micellar system based on a PEG-b-PLL copolymer and a peptide (Cys-Ile-Gln-Pro-Phe-Tyr-Pro, CP7) for receptor-mediated endocytosis. Using this peptide, the CP7-PEG-b-PLL nanocarrier considerably enhanced the chemotherapeutic efficiency of doxorubicin compared with the free drug [90].

### 4.4. Anti-Inflammatory Activity

Bioactive peptides are frequently used to treat inflammation. Their anti-inflammatory effects involve modulating the release of inflammatory mediators; regulating key signaling pathways such as NF-κB, MAPK, and JAK-STAT; and decreasing oxidative stress reactions to curb inflammation development [91].

**Figure 10:**
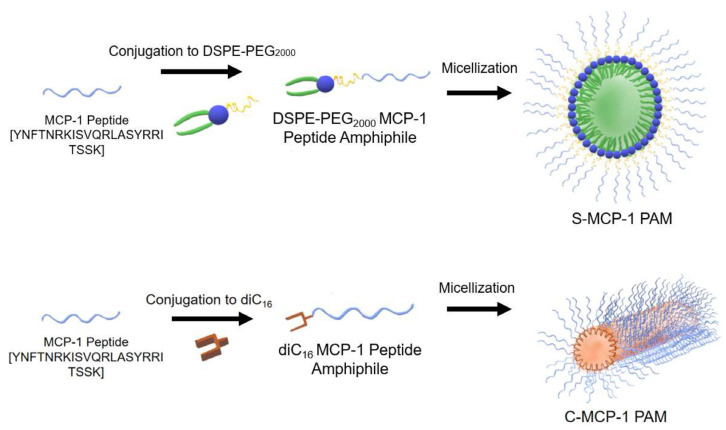
The monocyte chemoattractant protein-1 (MCP-1) peptide was conjugated to 1,2-distearoyl-sn-glycerol-3-phosphatidyl-ethanolamine (DSPE)–poly(ethylene glycol) (PEG2000) to form spherical MCP-1 peptide amphiphile micelles (PAMs) or to diC16 tail to form cylindrical MCP-1 PAMs [91].

Considering that monocyte chemoattractant protein-1 (MCP-1) plays a crucial role in the proliferation of monocytes, inflammation, and the pathogenesis of many diseases, spherical and cylindrical MCP-1 PAMs (peptide amphiphile micelles) based on DSPE-PEG2000 and diC16 PAMs have been prepared (Figure 10). MCP-1 is part of the family of C−C chemokines that promote monocyte and macrophage migration to sites of inflammation [4]. MCP-1 PAMs can target monocytes by incorporating the first peptide loop (residues 13–35) of MCP-1, which was previously found as the binding sequence of the CCR2 receptor and possesses chemotactic abilities [91]. Cylindrical PAMs showed a greater ability to attract monocytes compared to spherical PAMs in a chemotaxis assay.

### 4.5. Immunomodulatory Activity

Vasoactive intestinal peptide (VIP) is a neuropeptide that can downregulate innate immune responses in antigen-presenting cells (APCs) by inhibiting pro-inflammatory cytokine secretion and reducing cell surface marker expression. While the bioactivity of VIP might be utilized to promote transplant tolerance, advancements in drug delivery are necessary due to its inherently limited cellular delivery capacity. One potential solution involves using peptide amphiphiles (PAs), lipidated peptides that can self-assemble into micelles in water, thereby enhancing cellular association [92]. VIP amphiphiles were found to influence the shape, size, and surface charge of micelles (VIPAMs) as well as their cytotoxicity and immunomodulatory effects. Specifically, it was found that cylindrical VIPAMs with aspect ratios of 1.5–150 and moderate positive surface charge were able to potentiate the bioactivity of VIP, limiting TNF-α secretion and MHC II and CD86 surface expression on APCs.

### 4.6. Anti-Diabetic Activity

**Figure 11:**
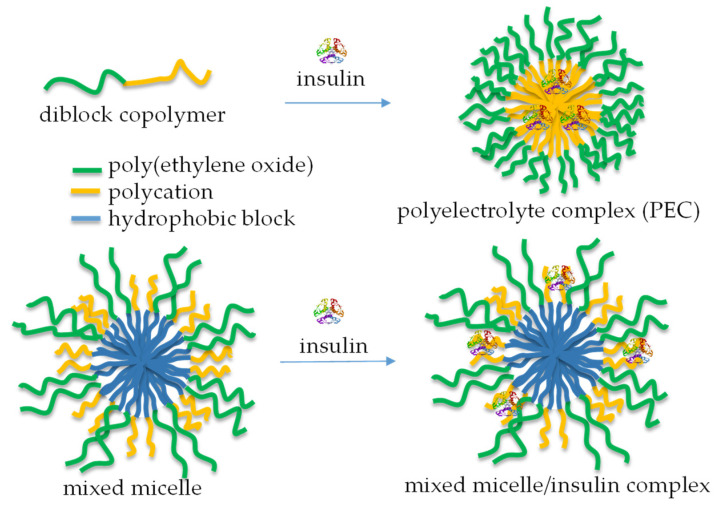
A schematic representation of the complexation of insulin with diblock copolymers comprising a polycation segment (**top**) and mixed polymer micelles, containing relatively short cationic segments in the shell (**bottom**).

Numerous bioactive peptides have demonstrated potential anti-diabetic properties and are promising as alternative treatment measures to prevent and manage diabetes [93]. The increasing interest in bioactive peptides as safer and more effective anti-diabetic agents has driven efforts to discover more peptide-based therapeutic candidates for managing diabetes [94]. In the case of proteins like insulin, incorporation into micellar nanocarriers allowed biomolecules to be protected from degradation. It facilitated uptake via transcellular and/or paracellular pathways, increasing therapeutic efficacy [95]. Typically, micelles are formed by mixing a cationic-neutral block copolymer and insulin in aqueous media (Figure 11, top) [96]. The formation of a polyelectrolyte complex (PEC) triggers the micellization process, yielding aggregates comprising a PEC core and a hydrated shell. Insulin-loaded polymeric micelles have also been prepared by cooperative self-assembly of poly(ethylene glycol)-b-poly(aspartic acid-co-aspartamidophenylboronic acid) and poly(aspartic acid-co-aspartglucosamine) copolymers [97]. The system showed glucose responsiveness under physiological conditions and offered self-regulated insulin delivery in response to physiological glucose levels.

Unlike PEC micelles, mixed micelles, comprising relatively long PEO (113 units) and very short cationic PDMAEMA (20 units) shell-forming blocks, were developed for the immobilization of insulin by complexation with PDMAEMA chains, without perturbing the function of PEO to act as a steric stabilizer (Figure 11, bottom). These micellar nanocarriers of insulin possessed superior colloidal stability and a sustained drug release profile [98].

## 5. Conclusions and Future Perspectives

Polymeric micelles exhibit low toxicity, excellent self-assembly ability, and biocompatibility, making them attractive for BP delivery. Micellar carriers are effective systems for enhancing the stability and bioavailability of BPs. Persistent research and development in this area hold the potential to overcome the limitations of traditional antibiotic therapies and provide new solutions for combating drug-resistant infections. Micellar carriers of peptides offer a promising approach for targeted and controlled delivery, leveraging the unique properties of peptides to enhance therapeutic outcomes. The interactions between carriers and peptide molecules can be tailored for controlled and sustained release by optimizing the compatibility between the peptide and polymers. Functionalizing the surface of polymeric micelles with targeting ligands also allows for the site-specific delivery of BPs. Typically, PMs are nanosized, allowing for easier distribution to target sites such as solid tumors. While the covalent attachment of BPs to micelles can provide stability, it may sometimes lead to peptide inactivation. Therefore, encapsulation or surface adsorption seems to be a more effective strategy for maintaining peptide activity. Designing micelles that respond to specific stimuli (e.g., temperature, pH) can allow for the controlled release of BPs, enhancing their therapeutic efficacy and reducing side effects.

Although micellar carriers are promising in preclinical studies, further research is needed to translate these findings into clinical applications. This includes addressing issues related to large-scale production, potential toxicity assessment, regulatory approval, cost-effectiveness, clinical trials, scale-up, and long-term stability experiments. The pharmacodynamics and pharmacokinetics, dosage regime, and side effects of micellar peptide carriers have not yet been sufficiently studied. Despite the advantages discussed, certain drawbacks of micellar peptide carriers include difficulties in the synthetic procedures and underdeveloped technologies for peptide loading. Guaranteeing high in vivo stability and bioavailability of BPs remains a critical challenge. To overcome these hurdles, further research focused on optimizing the design and functionalization of micellar carriers is needed.

Currently, multifunctional micelles are considered the most advanced delivery systems for BPs, capable of releasing their payload in response to specific triggers (pH, temperature, or enzymatic reactions), thus significantly enhancing the precision and efficacy of therapy. The synthesis of such complex carriers needs fine-tuning of micellar characteristics such as size, dispersity, morphology, drug-loading efficiency, biodegradability, stimuli responsiveness, interaction with cells, and internalization, among others. Micellar carriers can also enhance the permeability of antiviral peptides across biological barriers, such as the gastrointestinal tract or blood–brain barrier, facilitating delivery to otherwise inaccessible sites. In the future, with ongoing advancements, more and more micellar systems will be successfully transferred to clinical use, revealing their potential to revolutionize many therapies by providing more effective and targeted treatment options.

## Figures and Tables

**Table 8:** Characterization techniques of micellar nanocarriers of BPs [4].

Characterization Technique	Characteristic
Dynamic and static light scattering (DLS and SLS)	Size (hydrodynamic radius; radius of giration) Particle size distribution (dispersity) Aggregation number (N_agg_)
Atomic force microscopy (AFM), scanning and transmission electronic microscopy (SEM and TEM, cryo-TEM)	Morphology Size
Fluorescence and surface tension	Critical micellar and association concentrations (CAC and CMC)
Electrophoretic Light Scattering (ELS)	Surface charge (zeta potential)
Differential scanning calorimetry (DSC), X-ray diffraction, Fourier transform infrared spectroscopy (FTIR), nuclear magnetic resonance (NMR)	Critical micellization temperature (CMT) Degree of crystallinity; drug/polymer interactions
UV spectroscopy, high-performance liquid chromatography (HPLC), liquid chromatography–mass spectroscopy analysis	Drug loading efficiency (DLE), drug encapsulation efficiency (DEE), release kinetics
Small-angle X-ray and neutron scattering (SAXS) (SANS)	Size Structural properties

## Data Availability

The data used during the current study are available from the corresponding author upon request.
